# Effect of COVID-19 vaccination on the menstrual cycle

**DOI:** 10.3389/fmed.2022.1065421

**Published:** 2022-12-16

**Authors:** Melissa Jung Chao, Carlo Menon, Mohamed Elgendi

**Affiliations:** ^1^Faculty of Medicine, University of British Columbia, Vancouver, BC, Canada; ^2^Biomedical and Mobile Health Technology Lab, Department of Health Sciences and Technology, ETH Zürich, Zurich, Switzerland

**Keywords:** COVID-19, menstruation, menstrual disturbance, menstrual change, pandemic, lockdowns

## Abstract

Numerous anecdotal accounts and qualitative research studies have reported on post-vaccination menstrual irregularities in women of reproductive age. However, none have quantified the impact. This is the first systematic review and meta-analysis to quantify and characterize the menstrual irregularities associated with vaccination for women of reproductive age. A search on July 20, 2022, retrieved articles published between December 1, 2019, and July 1, 2022, from MEDLINE, Embase, and Web of Science. The included articles were studies with full texts written in English that reported on menstrual irregularities for vaccinated vs. unvaccinated women of reproductive age. The quality of the studies was evaluated using the Study Quality Assessment Tool for Observation Cohort and Cross-Sectional Studies. Four observational studies were included. Review Manager was used to generating a forest plot with odds ratios (ORs) at the 95% confidence interval (CI), finding statistically significant associations between vaccination and menstrual irregularities for 25,054 women of reproductive age (OR = 1.91, CI: 1.76–2.07) with a significant overall effect of the mean (*Z* = 16.01, *p* < 0.0001). The studies were heterogeneous with significant dispersion of values (χ^2^ = 195.10 at *df* = 3, *p* < 0.00001, *I*^2^ = 98%). The findings of this systematic review and meta-analysis are limited by the availability of quantitative data. The results have implications for treating women of reproductive age with menstrual irregularities and informing them about the potential side effects of vaccinations.

## Introduction

Vaccines that protect against infection of severe acute respiratory syndrome coronavirus 2 (SARS-CoV-2), the virus that causes the novel coronavirus disease 2019 (COVID-19), were first developed in early 2020. Early reports have described general side effects, but only recently has the media been reporting about menstrual side effects for women of reproductive age. Despite the media coverage, population-level evidence linking COVID-19 vaccination to menstrual cycle characteristics is limited.

As of late 2020, the United States Center for Disease Control and Prevention (CDC) has declared that COVID-19 vaccines are safe and effective at protecting people from severe illness, hospitalization, and death (1). Further research indicates that the COVID-19 vaccines may reduce the likelihood of SARS-CoV-2 infection upon exposure, reduce the likelihood of symptoms upon infection, and reduce the infectiousness of individuals with COVID-19 (2).

As of September 25, 2022, over 615 million cumulative COVID-19 cases worldwide resulted in over 6.5 million deaths (3). Since over 12 billion COVID-19 vaccine doses have been administered, large proportions of the world have received vaccinations at unprecedented rates (3). Of particular interest, women of reproductive age, including women who are pregnant, breastfeeding, trying to become pregnant, or may soon become pregnant, are encouraged to get vaccinated by the CDC (4).

For the general population, typical post-vaccination side effects include tiredness, headache, muscle pain, chills, fever, and nausea; at the location of the injection, pain, redness, and swelling have also been reported (5). For women of reproductive age, vaccine-derived menstrual cycle irregularities may be a potential side effect. However, clinical trials and the Vaccine Adverse Event Reporting System do not collect data on menstrual cycle side effects, so this potential side effect is currently not reported (6).

Menstrual cycle regularity is an important physiological process in the health of women of reproductive age, which correlates with general health and fertility (7). The typical menstrual cycle ranges from 26 to 35 days, with menses lasting 5 days (8). Regular menstrual patterns may include sporadic or stress-induced perturbances, which can result in changes to or a missed cycle (9). Furthermore, exposure to a variety of factors may influence a woman's menstrual cycle. These can include lifestyle factors (such as stress, shift work, exercise, dietary composition, and smoking/drugs/alcohol intake), life history factors (such as age at menarche and parity), environmental factors (such as air pollution and endocrine disrupting chemicals), and biological factors (such as body weight, age, ovarian conditions, and genetics) (10).

The menstrual cycle is regulated by circulating sex hormones along the hypothalamic-pituitary-gonadal axis (HPG axis), which ultimately controls the shedding of the endometrium tissue during menstruation (11). The hypothalamus and pituitary are also responsible for corticotropin-releasing hormone (CRH) and subsequent cortisol hormone production, a steroid hormone that is produced in response to stress, inflammation, and immune functions (11). Upon exposure to stressful stimuli and CRH secretion, CRH triggers an inflammatory action in the receptors in female reproductive organs that are involved in ovulation and degradation of the corpus luteum (11). CRH and cortisol reduce female sex hormone levels, resulting in menstrual abnormalities upon exposure to stressful stimuli (11).

Unfortunately, questions about post-vaccination menstrual symptoms have been excluded from vaccine trials, so the impact of vaccination on women of reproductive age is currently unclear. Various characteristics, including the prevalence of such menstrual changes, how long these changes have persisted, the expected fluctuations in menstrual cycling, and the impact of the vaccination on menstrual irregularities, are currently under-researched (6).

As the COVID-19 pandemic continues, women of reproductive age continue to seek vaccination to protect against severe disease and hospitalization. As such, the vaccination schemes may present reproductive and sexual health challenges for women of reproductive age. Although recent reviews have qualitatively described post-vaccination menstrual abnormalities for women of reproductive age, none have sought to quantify the potential impact of COVID-19 vaccination on menstrual irregularities (6, 12, 13). To our knowledge, this is the first systematic review and meta-analysis to review and quantify the impact of COVID-19 vaccinations on the menstrual cycles of women of reproductive age and to recommend future avenues of study.

## Methods

This systematic review and meta-analysis were conducted according to the guidelines presented in the Preferred Reporting Items for Systematic reviews and Meta-Analyses (PRISMA) (14, 15). A search protocol was developed in consultation with a librarian from the University of British Columbia. The protocol was not registered. The review process consisted of five phases: (1) potential articles were identified through database searches and manual searches, (2) articles were assessed for eligibility in adherence to the inclusion and exclusion criteria, (3) quality appraisal of the included articles was performed using the National Institute of Health (NIH) Study Quality Assessment Tools, (4) data on outcomes of interest from the included articles were extracted, and (5) data analysis and statistical analysis were performed on the extracted data (16).

### Database and manual searches for potential articles

After consulting with a librarian from the University of British Columbia, search terms were developed to reflect an interest in COVID-19, menstruation, and vaccination. The COVID-19 search included the following terms: COVID-19, SARS-CoV-2, nCoV-2019, coronavirus infection, viral pneumonia, and pandemics. The menstruation search terms included menstruation, menstruation disturbance, menses, menstrual flow, menstrual discharge, menorrhea, menarche, and monthlies. Search terms that encompassed vaccination included vaccination, vaccines, and COVID-19 vaccines. The full search terms are available in Supplementary method 1.

The search terms were deployed on July 20, 2022, to identify peer-reviewed observational articles published between December 1, 2019, and July 1, 2022, from MEDLINE, Embase, and Web of Science (17–19). Following the database search, the reference lists of relevant eligible articles were manually searched to identify additional eligible articles.

### Article review according to the inclusion and exclusion criteria

Following the article search, all potential articles were compiled and uploaded into the Covidence tool for systematic reviews, where duplicates were automatically removed (20). The articles were filtered according to the inclusion and exclusion criteria during this process. Subsequently, the article review phase consisted of a title and abstract screening, and a full-text review. This process was completed in a blind, independent manner by MC and ME to avoid selection bias. Disagreements were discussed until a consensus was reached.

### Inclusion and exclusion criteria

The inclusion criteria were: (i) articles published from December 1, 2019, to July 1, 2022, found in MEDLINE, Embase, and Web of Science, (ii) original articles that described observational studies, (iii) articles that had full-text available in English, (iv) articles that discussed menstruating women of reproductive age, and (v) articles specifically reporting on menstrual cycle irregularities for unvaccinated and COVID-19 vaccinated women of reproductive age (17–19). However, articles were not excluded based on geographical location, patient age, COVID-19 status, pregnancy status, ovulatory status, reproductive history, vaccine type, other vaccination-related symptoms, or any other patient factors.

The exclusion criteria were: (i) articles that did not have abstracts, (ii) articles that did not have a full-text available in English, (iii) publication types that were opinion pieces, letters, commentaries, guidelines, or simulations/models, (iv) articles that did not discuss women of reproductive age, and (v) articles that did not discuss menstrual cycle irregularities for COVID-19 vaccinated and unvaccinated women of reproductive age.

### Quality appraisal of the cross-sectional studies

The 14-item, NIH Study Quality Assessment Tool for Observation Cohort and Cross-Sectional Studies was used to critically appraise the cross-sectional studies included in this systematic review and meta-analysis (16). This tool was specifically designed to critically appraise cross-sectional studies for a systematic review. Thus, in this systemic review, this tool was used to assess the internal validity of the study design of each included cross-sectional study (16).

The NIH assessment tool consists of 14 questions that were applied to each study. Specifically, the tool assessed the clarity of the research question, the clarity of the study population, the participation rate of eligible persons, if the participants were recruited from the same population, the justification for sample size, if the exposures of interest were measured before the outcome of interest was measured, the sufficiency of the timeframe, how variable amounts of exposure impact the outcome, the validity of the exposure measurements if the exposures were measured multiple times, the validity of the outcome measured, if the assessment was blinded if the attrition rate to follow-up was <20%, and how the impact of the potential confounding variables was measured (16).

For each of the 14 items, each cross-sectional study was scored as Yes, No, or Not applicable (16). For each article, the number of items that reduced the bias was totaled. The articles were then categorized into quartiles to represent their risk of bias. The first quartile consisted of articles that met 11–14 of the NIH criteria, which represented low risk of bias. The second quartile consisted of articles that met 7–10 of the NIH criteria, which represented some risk of bias. The third quartile consisted of articles that met 4–9 of the NIH criteria, which represented moderate risk of bias. The fourth quartile consisted of articles that only met 0–3 of the NIH criteria, which represented high risk of bias. Only cross-sectional studies that fell within the first quartile were included in this systematic review. The appraisal tool was completed by MC and ME in a blind and independent manner, and any disagreements were discussed until resolved.

### Quality appraisal of the cohort studies

The NIH Study Quality Assessment Tool for Observation Cohort and Cross-Sectional Studies was also used to appraise the cohort studies included in this systemic review and meta-analysis (16). This tool was specifically designed to critically appraise cohort studies for a systematic review. Thus, in this systemic review, this tool was used to assess the internal validity of the study design of each included cohort study (16).

As previously noted, this 14-item quality assessment tool consists of 14 questions applied to each study. Specifically, the tool assessed the clarity of the research question, the clarity of the study population, the participation rate of the eligible persons, if the participants were recruited from the same population, the justification for sample size, if the exposures of interest were measured before the outcome of interest was measured, the sufficiency of the timeframe, how the variable amounts of exposure related to the outcome, the validity of the exposure measurements, if the exposures were measured multiple times, the validity of the outcome measured, if the assessment was blinded, if the attrition rate to follow-up was <20%, and how the impact of potential confounding variables was measured.

For each of the 14 items, each cross-sectional study was scored as Yes, No, or Not applicable (16). For each article, the number of items that reduced the bias was totaled. The articles were then categorized into quartiles to represent their risk of bias. The first quartile consisted of articles that met 11–14 of the NIH criteria, which represented low risk of bias. The second quartile consisted of articles that met 7–10 of the NIH criteria, which represented some risk of bias. The third quartile consisted of articles that met 4–9 of the NIH criteria, which represented moderate risk of bias. The fourth quartile consisted of articles that only met 0–3 of the NIH criteria, which represented high risk of bias. Only cross-sectional studies that fell within the first quartile were included in this systematic review. The appraisal tool was completed by MC and ME in a blind and independent manner, and any disagreements were discussed until resolved.

### Data extraction of outcomes of interest

During the data extraction phase, the reviewers generated a 30-item Covidence data collection form, populated each of the 30 items for all included articles, and exported the populated form into Microsoft Excel (20, 21). The data collection form captured the study characteristics and the outcomes of interest. Outcomes of interest were extracted from both the included article and its corresponding Supplementary information files.

The outcome of interest was the incidence of irregularities in menstrual cycles. The research question was to investigate the incidence of menstrual irregularities in women of reproductive age following COVID-19 vaccination compared to unvaccinated women of reproductive age. In this context, irregular menstrual cycles consisted of self-reported changes in menstrual volume, menstrual duration, cycle length, ovulation timing, and/or pain level.

Discrete numerical data were collected on the incidence of menstrual irregularities for women of reproductive age following COVID-19 vaccination in comparison to the rate for unvaccinated women of reproductive age. To manage the unpopulated data fields, the number of women was imputed based on the given values. If, for example, the number of vaccinated women with menstrual irregularities was missing, while the total number of women with menstrual irregularities and the number of unvaccinated women with menstrual irregularities were given, the missing value was calculated using subtraction.

Data collection was performed irrespective of dose numbers, such as first dose, second dose, or booster dose(s). For articles that did include the dose number, the first dose was used because many patients received the first dose but not subsequent doses. Furthermore, this systematic review did not provide information on the resolution of menstrual disruptions based on the vaccine manufacturer.

### Statistical analysis

The odds ratio (OR) at the 95% confidence interval (CI) was calculated for each included article to determine the odds that the outcome is associated with the exposure. The Mantel-Haenszel fixed-effect method was employed to assess the discrete data. Subsequently, the *I*^2^-, τ^2^-, and χ^2^-test were employed to quantify the level of heterogeneity between studies in the meta-analysis, and a *p*-value was calculated to determine the level the significance of the heterogeneity.

Weighted mean differences were used to analyze statistical data effectiveness, and a 95% CI was calculated. Once the population mean OR was determined, the *Z*-test statistic was employed to evaluate the reduction of uncertainty in past events, and the *p*-value was calculated.

## Results

### Search results

A total of 84 articles were identified through a search of three databases (14 articles from MEDLINE, 68 articles from Embase, and two articles from Web of Science) (17–19). After 16 duplicate articles were removed, the abstracts and titles of the remaining 71 articles were screened, resulting in 16 articles for full-text review. Following the full-text review, one article remained for data extraction. All the included articles were hand-searched for references; thus, three additional articles were identified for review.

**Figure 1:**
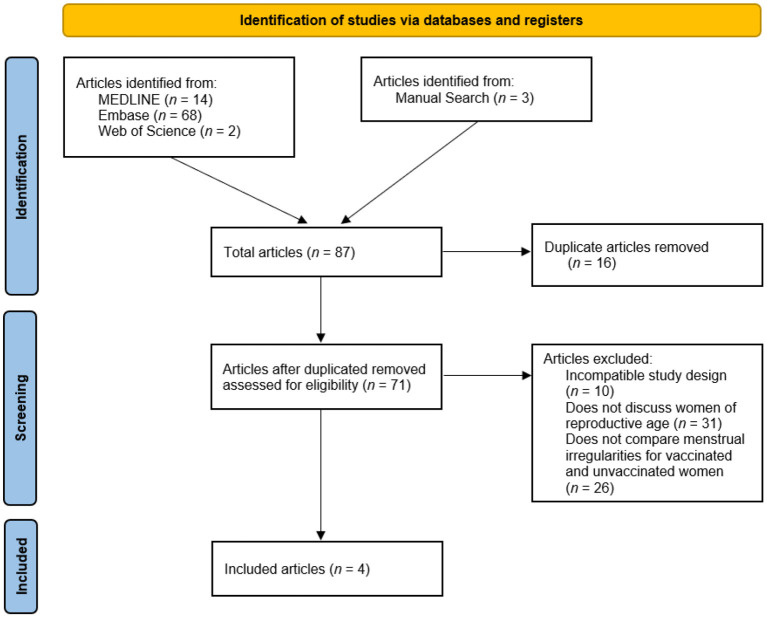
PRISMA flow diagram for systematic reviews.

Consequently, four articles were included in this systematic review and meta-analysis, as described in Figure 1. They described 19,019 women of reproductive age in the vaccinated (exposed) group and 6,045 women of reproductive age in the unvaccinated (unexposed) group, for a total of 25,054 women.

### Quality assessment results

All four articles included in this systematic review fell into the first quartile during the screening process, where cohort studies were reviewed using the NIH Study Quality Assessment Tools (16). These articles had different observational study designs: two cross-sectional and two cohort studies. Of the four articles, there is low representation from both high-income countries (two articles from the United States) and middle-income countries (one from Jordan and one from China); there is no representation from low-income countries.

The Study Quality Assessment Tool scores for all the included articles are presented in Table 1. The full quality assessment is presented in Supplementary Tables 1, 2.

**Table 1:** Characteristics of all included articles.

**Article ID**	**Author**	**Year**	**Title**	**Country**	**Study design**	**Sample size**	**Age in years (mean, median)**	**Quality assessment tool for observational cohort and cross-sectional studies (16)**
1	Edelman et al. (7)	2022	Association between menstrual cycle length and coronavirus disease 2019 (COVID-19) vaccination: a U.S. Cohort.	United States of America	Cohort	3,959	N/A, N/A	12/14
2	Muhaidat et al. (22)	2022	Menstrual symptoms after COVID-19 vaccine: a cross-sectional investigation in the MENA region.	Jordan	Cross-section	2,269	34.3, N/A	11/14
3	Wesselink et al. (23)	2022	A prospective cohort study of COVID-19 vaccination, SARS-CoV-2 infection, and fertility	United States of America	Cohort	N/A	N/A, N/A	11/14
4	Zhang et al. (24)	2022	COVID-19 vaccine and menstrual conditions in female: data analysis of the vaccine adverse event reporting system	China	Cohort	14,431	N/A, 35	11/14

That N/A means not applicable.

### Irregular menstrual cycle after vaccination

**Figure 2:**
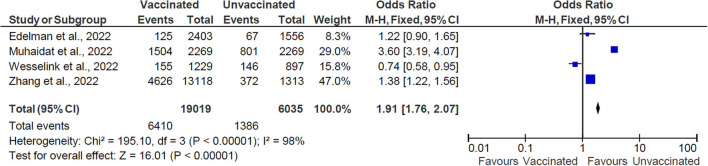
The effect of vaccination on menstrual cycle changes. M-H, Mantel-Haenszel method; CI, confidence interval; and df, degrees of freedom.

Overall, 25,054 women of reproductive age were included in the studies in this systematic review, consisting of 19,019 vaccinated women and 6,035 unvaccinated women of reproductive age (Figure 2). The OR with the accompanying 95% CI was calculated for each of the values ranging from 0.74 to 1.38. Only one study had an OR value <1 (23).

The pooled OR is 1.91 (CI: 1.76–2.07), indicating an association between vaccination and menstruation changes. The statistical analysis results indicate heterogeneity between the studies and the dispersion of values is statistically significant (χ^2^ = 195.0 with *df* = 3, *p* < 0.00001, *I*^2^ = 98%). The overall effect of the mean was statistically significant (*Z* = 16.01, *p* < 0.0001).

## Discussion

In most recent academic articles, the investigation has focused on the profound impacts of COVID-19, including extensive media discussion of COVID-19 vaccines causing menstrual cycle irregularities. During such unprecedented situations, many confounding variables complicate research on this topic, including significant lifestyle changes, stress, vaccination, and COVID-19 disease (12). Because these factors may significantly influence the results of the research, it is difficult to determine if menstrual cycle fluctuations are due to the impact of the exposure rather than the confounding variables caused by the pandemic.

First, the stress of the COVID-19 pandemic, especially the associated disruptions to daily life, such as lockdowns, has been reported to influence the HPA axis, thus contributing to variations in menstrual cycles (25). However, the effects were temporary as the menstrual cycle returned to its expected time during the next cycle (26). Additionally, dexamethasone, a corticosteroid used to treat hospitalized COVID-19 patients, is a risk factor for menstrual irregularities. This drug is thought to act *via* endometrial cortisol, which may act on endometrial blood vessel maturation, leading to potential changes in menstrual blood loss and cycling (12, 27).

Similarly, recent research has reported a correlation between SARS-CoV-2 infection and changes in the menstrual cycle length and volume; in particular, one systematic review observed decreases in menstrual volume and extended menstrual cycles (12). However, it reported no impact of COVID-19 disease severity on menstrual cycle changes (12). Recent studies have indicated that the use of oral estrogen-containing contraceptives by women of reproductive age reduces the risk of post-vaccination menstrual irregularities (28). This has been attributed to increased concentrations of circulating estrogen and progesterone, which play an anti-inflammatory and immunomodulatory role to protect against severe COVID-19 disease outcomes (29).

One article found that vaccination was associated with a delay in the next menstrual cycle for individuals not taking hormonal contraceptives, compared to pre-vaccine cycles, for an average cycle delay of 2.3 days (26). However, the timing of the vaccination relative to the woman's menstrual cycle did not have a definitive impact on the flow of the subject's subsequent menstrual period (26). Finally, this article found that the brand of the vaccine was not associated with changes in the cycle timing or flow of the participants' subsequent menstrual period (26).

Additionally, a cross-sectional study conducted in Spain examined the self-reported menstrual characteristics of 14,153 women following full course vaccination (30). That study reported that the most predominant premenstrual characteristics were increased irritability, increased abdominal bloating, and greater fatigue; the most frequently reported post-vaccination menstrual changes were delayed menstruation and more menstrual pain and bleeding (30).

Despite evidence in support of short-term menstrual changes, menstrual cycles have been reported to return to normal after a few months with no long-term impacts (7, 31). Additionally, there is no research to support that the COVID-19 vaccinations impact fertility and long-term reproductive health; thus, they continue to be recommended for women of reproductive age (13, 31).

Recently, two biological pathways may explain vaccination-derived immune stimulation of the menstrual system (32). First, innate immune cells may transiently interfere with the reproductive hormones that subsequently cause prolonged cycling (26). To support this, vaccination administered during the follicular phase of the menstrual cycle produced immune cell-mediated hormonal changes, which prolonged the follicular phase in one research study (33). As mentioned previously, this mechanism is further supported when women are administered estrogen- and progesterone-containing hormonal contraceptives, which was shown to interfere with natural hormonal cycling (26). The second mechanism involves macrophage and natural killer cell interference with the breakdown and regeneration of tissue in the uterus (32). These immune cells mediate tissue repair and breakdown in the uterus throughout the menstrual cycle (32). The relationship between menstrual flow and age supports this hypothesis. Older women have less effective repair systems (consisting of macrophages and natural killer cells) in the endometrial lining, causing higher menstrual flow rates; as immune systems are associated with menstrual flow, this suggests a role of immune cells in the observed increase in menstrual flow (34). Taken together, these two mechanisms may be responsible for both increased cycle length and increased menstrual volume (32).

Because the current evidence on this topic is limited, this systematic review and meta-analysis agree with the information presented in widespread social media reports and contribute to the discussion. Despite initial evidence regarding the association between COVID-19 vaccinations and menstrual irregularities, clinicians frequently encounter women of reproductive age who experience changes in their menstrual cycle. These findings will contribute to a growing body of evidence surrounding potential changes in menstrual cycles that will cumulatively inform clinicians about how to treat patients.

### The context of vaccine hesitancy

These findings are relevant to the worldwide trend of vaccine hesitancy. There is concern that post-vaccination menstrual irregularities are a source of vaccine hesitancy for this population and may provide fuel for anti-vaccination campaigns (26). The findings of this meta-analysis are relevant to the greater context of vaccine acceptance and designing strategies to increase uptake.

High heterogeneity in vaccination uptake rates exists between countries. The health system is reported to be a strong determinant of vaccine uptake as they are responsible for delivery and effective communication to the public (35). Other major determinants (36) include:

- Differences in local norms and cultures,
- Exposure to credible media sources,
- Awareness and severity of COVID-19, and
- Access to healthcare services.

Interestingly, Asian countries have strong trust in government recommendations and high vaccine uptake, while middle-income countries tend toward acceptance of vaccines (35).

There is a mixed response to encouraging vaccination uptake through vaccine mandates (37). Public health agencies must balance the goal of herd immunity with the freedom to choose vaccination (37). In 2021, the European Union produced principles that state that vaccination must occur according to individual choice, so an individual must consider the benefits against the harms but face no discrimination for refusing to be vaccinated (37). Considering their principles, the policies across the European Union vary in terms of vaccination requirements, passports, mask mandates, and testing requirements (37).

Significant gains globally will occur by targeting populations of low income and low education (35, 36). Hesitancy can stem from marginalization, social exclusion, negative experiences with healthcare, misinformation, mistrust in government, and mistrust in health authorities (38). Strategies to address these populations include providing clear communication using a trusted local source, addressing historical issues of distrust, and being sensitive to religious or cultural beliefs (35). Interestingly, many cultures are more accepting of getting vaccinated when recommended by an employer but are less likely to get vaccinated if mandated by an employer; such attempts are perceived as limiting the employee's freedom or expressing employers' self-interest (35).

Many personal factors are associated with high vaccine uptake, including strong COVID-19 knowledge, high perceived seriousness of the pandemic, and good preventative practices; these patterns were consistent in many low- and middle-income countries, including Congo, Ethiopia, and some middle eastern populations (36).

There is a vital role for health workers in building confidence and acceptance of vaccines in public; clear communication about the safety and efficacy of the COVID-19 vaccine, as well as personal reasons and recommendations from a trusted health worker, can increase vaccine uptake in many populations (38).

Populations with specific health concerns, like pregnant women, often receive poor information regarding the benefits and safety of vaccinates (39). They are more likely to accept a vaccine from a health worker when health workers educate patients about it and recommend it to them, so vaccine counseling should be part of prenatal and pregnancy care. Similarly, health workers have a crucial role in communicating the safety and efficacy of vaccines despite the short-term menstrual side effects to women of reproductive age; they may attempt to provide personal recommendations to encourage vaccination to this population (31).

These strategies can help limit the consequences of the COVID-19 pandemic and improve vaccine uptake. Vaccines remain the most effective manner to limit the morbidity and mortality associated with SARS-CoV-2 infection (38). Research has shown that mass vaccination to achieve herd immunity is the most effective manner to overcome the COVID-19 pandemic (37). It is worth reiterating that despite short-term menstrual changes, there is no research to support the fear that COVID-19 vaccinations impact fertility, and vaccines are recommended for women of reproductive age (13, 31).

### Limitations of the review

There are several limitations associated with this systematic review and meta-analysis. The primary limitation is the lack of randomized control trials, which restricts the investigation of a potential causal link (6). As such, the included articles discussing observational studies may only provide support for a correlative link.

Second, the increased frequency of menstrual irregularities reported in the media creates a heightened awareness of health, especially menstrual health, due to the increase in reporting during the pandemic and anecdotal reports of vaccination side effects (6). This has caused many women of reproductive age to monitor their health more closely, which may result in false attributions or overreporting.

Third, menstrual characteristics are often subjective, and data are often collected using self-reporting methods, leaving room for miscalculation and systemic error (6). Many methods exist to monitor a woman's menstrual cycle, so it is unclear what methods the women in the included articles used to quantify their irregularities. In particular, the symptom of pain is a subjective measurement, and heightened awareness of symptoms due to media communication could produce higher pain scores (6).

Fourth, there were limited amounts of population data so subgroup analyses could not be performed. Due to the lack of reporting, the type of vaccine manufacturer could not be correlated with menstrual irregularities, which may reduce the applicability in real-world decisions to select one vaccine over another. Similarly, this systematic review could not account for the number of vaccine doses that a subject received, which may be further complicated as boosters continue to be administered at rapid rates. Data were further limited in the study's inability to report when a menstrual irregularity was observed relative to when the vaccine was administered. Such factors may include the length of time after menstruation in which the woman received the vaccine, the phase of the woman's menstrual cycle when she received the vaccine, and if multiple doses were taken in the same cycle, which may provide further clarity into the temporal aspect of the vaccine's impact on menstrual regularity.

Fifth, this systematic review's strict inclusion criteria limit the generalizability of its findings to the global population. Searches were limited to published articles available in English, which primarily produced articles from high-income countries and middle-income countries, with no representation from low-income countries. This may favor reporting specific subpopulations (including some racial groups) that may have variations not captured in this review, so our results may not represent the global population.

Finally, there were reporting biases in the selected articles because all the articles were observational studies, which are known to overreport cases in the exposed group and underreport normal cases, causing an overestimating of the effect size.

### Future work

To overcome the lack of resolution by vaccine type, future articles may investigate the relationship between the vaccine manufacturer, the number of vaccine doses, and menstrual irregularities. As booster shots become more prevalent, it is crucial to investigate their impact. Additional studies may record the time it takes for menstrual irregularities to normalize, such that subsequent review articles may synthesize the results to inform practitioners of the full scope of these side effects.

Future studies may investigate the co-occurring impacts of SARS-CoV-2 infection with COVID-19 vaccination to fully characterize the impact on women of reproductive age. Future studies should also consider how menstrual cycle irregularities may have downstream impacts on women's reproductive and sexual health.

To overcome reporting bias, future studies should investigate unpublished, non-English literature from a representative selection of countries. Moreover, understanding the heterogeneity between studies will help elucidate how a broader context may bias the results.

## Conclusion

Overall, this systematic review synthesized the growing body of evidence surrounding the impact of the COVID-19 vaccination on menstrual cycles, finding an association between COVID-19 vaccination and menstrual irregularities. These findings have implications for clinicians in treating the menstrual concerns of women of reproductive age and informing them of the potential side effects of vaccination. Despite evidence that supports short-term menstrual changes, menstrual cycles return to normal a few months after being vaccinated, and there are no reported long-term impacts. Thus, vaccination continues to be recommended for women of reproductive age. Additional research is needed to characterize these women's wide variety of menstrual experiences.

## Data availability statement

The original contributions presented in the study are included in the article/Supplementary material, further inquiries can be directed to the corresponding author.

## Author contributions

MC, CM, and ME conceived the study. ME designed the study. All authors approved the final manuscript.
