# Erratum: Effect of COVID-19 vaccination on the menstrual cycle

**DOI:** 10.3389/fmed.2023.1170876

**Published:** 2023-03-02

**Authors:** Frontiers Production Office

**Affiliations:** Frontiers Media SA, Lausanne, Switzerland

**Keywords:** COVID-19, menstruation, menstrual disturbance, menstrual change, pandemic, lockdowns

An omission to the funding section of the original article was made in error. The following sentence has been added: “Open access funding was provided by ETH Zurich.”

The original article has been updated.

