# Supplementary material for: Effect of COVID-19 vaccination on the menstrual cycle
Source: Front Med (Lausanne). 2022 Dec 16;9:1065421. doi: 10.3389/fmed.2022.1065421 (PMC9802578; doi:10.3389/fmed.2022.1065421)
Supplement: Supplementary file 1 [file Data_Sheet_1.docx]

**SUPPLEMENTARY METHOD 1**

Supplementary Method 1: OVID Search Terms

{(Coronavirus infection/ OR COVID-19/ OR SARS-CoV-2/ OR pneumonia, viral/ OR pandemics/ or coronavirus/) OR [(COVID-19 OR coronavirus* OR SARS-CoV-2 OR nCoV-2019 OR viral pneumonia* OR pandemic* OR coronavirus*).mp. (mp = title, abstract, original title, name of substance word, subject heading word, floating sub-heading word, keyword heading word, organism supplementary concept word, protocol supplementary concept word, rare disease supplementary concept word, unique identifier, synonyms)]} AND {(Exp Menstrual Disturbances/ or exp Menstruation/ or exp Menarche/) OR [(menstrua* or menstrual disturbance* OR menstrual change* OR menstrual flow* OR menstrual discharge* OR menses* OR menorrhea* OR menorrhoea* OR menarche* or monthlies*).mp. (mp=title, abstract, original title, name of substance word, subject heading word, floating sub-heading word, keyword heading word, organism supplementary concept word, protocol supplementary concept word, rare disease supplementary concept word, unique identifier, synonyms)]} AND {(vaccination/ OR vaccines/ OR COVID-19 vaccines/) OR [(vaccination* OR vaccine* OR COVID-19 vaccine*)*).mp. (mp=title, abstract, original title, name of substance word, subject heading word, floating sub-heading word, keyword heading word, organism supplementary concept word, protocol supplementary concept word, rare disease supplementary concept word, unique identifier, synonyms)]}
