# Supplementary material for: Effect of COVID-19 vaccination on the menstrual cycle
Source: Front Med (Lausanne). 2022 Dec 16;9:1065421. doi: 10.3389/fmed.2022.1065421 (PMC9802578; doi:10.3389/fmed.2022.1065421)
Supplement: Supplementary file 3 [file Table_2.docx]

**SUPPLEMENTARY TABLE 2**

Supplementary Table 2: Johanna Briggs Institute Critical Appraisal Checklist for Quasi-Experimental Studies

| **Article Number** | **1** |
| --- | --- |
| **Criteria** |  |
| 1. Is it clear in the study what is the ‘cause’ and what is the ‘effect’ (i.e. there is no confusion about which variable comes first)? | 1 |
| 2. Were the participants included in any comparisons similar? | 1 |
| 3. Were the participants included in any comparisons receiving similar treatment/care, other than the exposure or intervention of interest? | 1 |
| 4. Was there a control group? | 0 |
| 5. Were there multiple measurements of the outcome both pre and post the intervention/exposure? | 0 |
| 6. Was follow up complete and if not, were differences between groups in terms of their follow up adequately described and analyzed? | 1 |
| 7. Were the outcomes of participants included in any comparisons measured in the same way? | 1 |
| 8. Were outcomes measured in a reliable way? | 1 |
| 9. Was appropriate statistical analysis used? | 1 |
| **Total** | 7 |
